# A systematic review and meta-analysis on the efficacy of vaccination against colibacillosis in broiler production

**DOI:** 10.1371/journal.pone.0301029

**Published:** 2024-03-22

**Authors:** Surya Paudel, Ilias Apostolakos, Ronald Vougat Ngom, Giuditta Tilli, Helena C. de Carvalho Ferreira, Alessandra Piccirillo

**Affiliations:** 1 Department of Infectious Diseases and Public Health, Jockey Club College of Veterinary Medicine and Life Sciences, City University of Hong Kong, Kowloon, Hong Kong SAR; 2 Clinic for Poultry and Fish Medicine, Department for Farm Animals and Veterinary Public Health, University of Veterinary Medicine Vienna, Vienna, Austria; 3 Veterinary Research Institute, Hellenic Agricultural Organization “DIMITRA”, Thessaloniki, Greece; 4 Department of Animal Production, School of Veterinary Medicine and Sciences, University of Ngaoundere, Ngaoundéré, Cameroon; 5 Veterinary Public Health Institute, Vetsuisse Faculty, University of Bern, Bern, Switzerland; 6 Department of Comparative Biomedicine and Food Science, University of Padua, Legnaro, Italy; 7 Flanders Research Institute for Agriculture, Fisheries and Food, Merelbeke, Belgium; Zagazig University Faculty of Agriculture, EGYPT

## Abstract

Colibacillosis, a disease caused by *Escherichia coli* in broiler chickens has serious implications on food safety, security, and economic sustainability. Antibiotics are required for treating the disease, while vaccination and biosecurity are used for its prevention. This systematic review and meta-analysis, conducted under the COST Action CA18217—European Network for Optimization of Veterinary Antimicrobial Treatment (ENOVAT), aimed to assess the efficacy of *E*. *coli* vaccination in broiler production and provide evidence-based recommendations. A comprehensive search of bibliographic databases, including, PubMed, CAB Abstracts, Web of Science and Agricola, yielded 2,722 articles. Following a defined protocol, 39 studies were selected for data extraction. Most of the studies were experimental infection trials, with only three field studies identified, underscoring the need for more field-based research. The selected studies reported various types of vaccines, including killed (n = 5), subunit (n = 8), outer membrane vesicles/protein-based (n = 4), live/live-attenuated (n = 16), and CpG oligodeoxynucleotides (ODN) (n = 6) vaccines. The risk of bias assessment revealed that a significant proportion of studies reporting mortality (92.3%) or feed conversion ratio (94.8%) as outcomes, had “unclear” regarding bias. The meta-analysis, focused on live-attenuated and CpG ODN vaccines, demonstrated a significant trend favoring both vaccination types in reducing mortality. However, the review also highlighted the challenges in reproducing colibacillosis in experimental setups, due to considerable variation in challenge models involving different routes of infection, predisposing factors, and challenge doses. This highlights the need for standardizing the challenge model to facilitate comparisons between studies and ensure consistent evaluation of vaccine candidates. While progress has been made in the development of *E*. *coli* vaccines for broilers, further research is needed to address concerns such as limited heterologous protection, practicability for application, evaluation of efficacy in field conditions and adoption of novel approaches.

## Introduction

Poultry meat is expected to hold a substantial share in global meat consumption, accounting for approximately half of the growth in meat production by 2032 [1]. As the poultry industry intensifies, ensuring optimum food safety and animal welfare becomes a top priority. However, bacterial pathogens, such as *Escherichia coli* pose a major challenge to the poultry industry [2]. *E*. *coli* is a gram-negative bacterium in the family of *Enterobacteriaceae* that normally resides in the healthy gut of chickens as a commensal [3, 4]. However, infection with pathogenic strains can lead to colibacillosis, a syndrome that affects chickens of all ages [5]. Colibacillosis in broilers can manifest in various clinical forms, including omphalitis, airsacculitis, femoral head necrosis and cellulitis, resulting in high condemnation rates and mortality [6–8]. The avian *E*. *coli* isolates are highly heterogenous, with pathogenicity likely involving coordination among several virulence genes, host factors or transfer of genetic elements among *E*. *coli* populations [9, 10]. Thus, understanding the pathogenesis of colibacillosis remains a challenge [11].

Colibacillosis is primarily treated with antibiotics. However, recent studies have shown the emergence of antibiotic-resistant strains of *E*. *coli* on a global scale [12]. Multidrug resistance in *E*. *coli* has become a concerning threat, even in flocks where no antibiotics are used [13–15]. In Europe, *E*. *coli* is identified as one of the most relevant antimicrobial resistant bacterial pathogens from poultry [16]. Consequently, there is a growing need to explore preventive strategies such as vaccination and biosecurity, as opposed to relying solely on antimicrobial treatments. In the past, numerous studies have reported a range of potential vaccine candidates as summarized in previous reviews and book chapters [5, 17–20]. However, there have been no systematic reviews conducted to assess the efficacy of *E*. *coli* vaccines in chickens. Such reviews are essential for providing clear, comprehensive evidence that can inform evidence-based recommendations. Consequently, this study, conducted under the framework of the COST Action CA18217—European Network for Optimization of Veterinary Antimicrobial Treatment (ENOVAT), aimed to carry out a systematic review and meta-analysis to understand the current evidence regarding the efficacy of vaccination in preventing colibacillosis in broiler chickens.

## Methods

This review was performed according to the Cochrane Handbook for Systematic Reviews of Interventions [21] and adheres to the structured and reporting guidelines outlined in the Preferred Reporting Items for Systematic Reviews and Meta-Analyses (PRISMA) 2020 [22].

### Protocol registration

A systematic review protocol was developed, registered in the University of Padua Research Archive institutional repository (https://hdl.handle.net/11577/3439974), and published on the Systematic Reviews for Animals and Food (SYREAF) website (https://syreaf.org).

### Eligibility criteria

The primary focus of this systematic review was to include controlled trials with natural disease exposure. However, disease challenge studies and observational studies were also considered. The studies had to be conducted in broiler production chain (Population) and assess the protective efficacy of vaccine candidates (Intervention). The vaccine intervention was compared to either an infected and untreated control group or a group that received placebo treatment (Comparator). The selected Outcomes of vaccine efficacy for this review were mortality, feed conversion ratio (FCR) and condemnation rate at the slaughterhouse. Articles written in English or Spanish were included, and no restrictions were imposed on publication date or geographical location of the studies.

### Sources of information

To ensure comprehensive coverage of articles, the following bibliographic databases for literature search were used that provide a high level of article recall in the biomedical field [23]: i) MEDLINE (via PubMed, https://pubmed.ncbi.nlm.nih.gov/), ii) CAB Abstracts (via Ovid, https://www.wolterskluwer.com/en/solutions/ovid/cab-abstracts-31), iii) Web of Science (WoS, http://webofknowledge.com/), and iv) Agricola (via ProQuest, https://www.proquest.com/). Searches in CAB Abstracts and Agricola were conducted through the University of Bern (Switzerland), while those in PubMed and WoS through the University of Padova (Italy). All databases of WoS were used, including WoS core collection, BIOSIS Citation Index, KCI-Korean Journal Database, Medline, Russian Science Citation Index and SciELO Citation Index. However, because of their research scopes, certain editions were excluded, namely Arts & Humanities Citation Index (A&HCI), Conference Proceedings Citation Index-Science (CPCI-S), Conference Proceedings Citation Index-Social Science & Humanities (CPCI-SSH) and Social Sciences Citation Index (SSCI). The initial search was conducted in September 2021 and a second search covering the period from September 2021 to October 2023 was performed in October 2023. The databases and search string were the same in both the search events.

### Search strategy and study selection

The search strategy employed a multi-stranded approach, utilizing various combinations of concepts to ensure comprehensive retrieval of relevant research and achieve high sensitivity [21]. The concept and the corresponding search strings are presented in Table 1.

**Table 1:** Bibliographic search strategy to identify studies examining the effect of vaccines against colibacillosis in broiler chickens.

Major terms	Key words
#1 Broilers	chicken* OR poultry* OR gallus OR broiler* OR flock
#2 Vaccination	vaccination* OR vaccine* OR bacterin* OR sub-unit* OR "killed vaccine*" OR "live vaccine*" OR "autogenous vaccine*"
#3 Colibacillosis	colibacillosis OR colisepticaemia OR peritonitis OR coli OR Escherichia OR coliform OR colisepticemia OR coligranuloma OR Hjarre’s OR "air sac disease" OR cellulitis OR osteomyelitis OR "brittle bone disease" OR salpingitis OR synovitis OR omphalitis OR enteritis OR "hemorrhagic septicemia" OR "chronic respiratory disease" OR "swollen head syndrome" OR "venereal colibacillosis" OR "coliform cellulitis" OR "yolk sac infection" OR APEC OR "pathogenic E. coli" OR "primary infection" OR "secondary infection" OR multifactorial OR multicausal
#1 AND #2 AND #3	records screened

The bibliographic records of the identified articles were downloaded in BibTeX format and imported to Rayyan [24]. To ensure data accuracy, deduplication process was conducted using the built-in function of Rayyan. The screening and evaluation of studies were conducted in two steps. In the first step, at least two independent reviewers screened titles and abstracts. Any conflicts or disagreements were resolved through discussion or with the involvement of a third reviewer. To maintain consistency among reviewers, a calibration exercise was first conducted by screening 25 randomly selected studies. The eligibility of studies was evaluated using a set of questions adapted from a previously published protocol [25] as:

1. Is the study original research assessing the use of vaccine(s) to prevent or control colibacillosis in broilers? YES [PASS], NO [EXCLUDE], UNCLEAR [PASS]
2. Does the study include an eligible comparator via a controlled trial, disease challenge study or observational study? YES [PASS], NO [EXCLUDE], UNCLEAR [PASS]

Studies were excluded only if all reviewers unanimously agreed that the answer to any of the screening questions was “no”. The studies that passed the first screening step proceeded to the next, where the full-text articles were retrieved and assessed for eligibility. In the second phase, the following set of questions was applied:

- Is a full text of more than 500 words available? YES [PASS], NO [EXCLUDE]
- Is a full text available in English and/or Spanish? YES [PASS], NO [EXCLUDE]
- Is the population of the study broilers? YES [PASS], NO [EXCLUDE]
- Is the intervention of the study the use of vaccine(s) to prevent or control colibacillosis in broilers? YES [PASS], NO [EXCLUDE]
- Is at least one of mortality, FCR, or condemnations at slaughter due to colibacillosis the outcome(s) described? YES [PASS], NO [EXCLUDE]
- Is the study design a controlled trial with natural disease exposure or a disease challenge study or an observational field study? YES [PASS], NO [EXCLUDE]

### Data extraction

A Microsoft Excel (2019 version) standardized spreadsheet, developed and validated by the authors, was used for data extraction. Relevant study characteristics, population type (broilers or broiler breeders), group size, year of the study, age of the birds during intervention and outcome assessment, and duration of observation were collected. Detailed data on the intervention were also extracted, including the vaccine type and commercial name, route and dose of administration, comparator group, unit of population, and total number of birds included. For studies involving disease challenge, information on challenge day, duration, strain, and administration route were collected.

Data extraction focused on mortality, FCR and condemnations at slaughterhouse due to colibacillosis. For mortality, the unit of measurement and assessment period were recorded. For studies reporting FCR and/or condemnations at slaughter, values such as FCR value and/or age/weight of slaughtered birds were extracted.

### Risk of bias

The risk of bias (RoB) assessment deviated from the original protocol and used instead a recently reported poultry-specific method [26]. Five domains of bias were evaluated, including bias from randomization (Domain 1), deviations in interventions (Domain 2), missing outcome data (Domain 3), measurement of the outcome (Domain 4), and selection of reported results (Domain 5). Briefly, each domain of bias is composed of several signaling questions that guide the overall risk of bias for each domain. This overall risk of each domain can then be reported as ‘‘low risk” ‘‘unclear”, or ‘‘high risk”. At the end, for each included study, a final overall risk of bias judgment is provided to each outcome based on the results from the five domains. Therefore, a ‘‘low risk of bias” outcome would result from all five domains being classified as ‘‘low risk”; ‘‘unclear” would result when either one or two domains for that outcome have been classified as ‘‘unclear”; and ‘‘high risk of bias” would result from at least three domains being classified as ‘‘unclear” or if at least one domain is classified as ‘‘high risk”. The RoB assessment was conducted only for mortality and FCR outcomes.

### Data synthesis and statistical analysis

The results of the literature search and selection were reported, and descriptive analysis of extracted data was done by using Microsoft Excel (version 2019). After data extraction, included studies were narratively summarized according to the type of vaccines used and study’s setting. The meta-analysis was performed using Revman version 5.4.1 according to Higgins et al. [21]. Considering the data of the selected studies, the meta-analysis was performed for two groups of vaccines (i.e. live attenuated vaccine and CpG oligodeoxynucleotides) with “mortality” as outcome. Some differences between included studies (age and route of inoculation, *E*. *coli* strain, dosage, etc.) were not considered during meta-analysis. The comparison concerned “infected and vaccinated” and “infected and no vaccinated” groups. Data used consisted of the number of dead animals per each group. The effect measure for outcome were odds ratios (with the 95% confidence levels) with a fixed model visualized through Forest plots. The heterogeneity among studies was evaluated with Cochrane test based on Chi-Squared. Significant heterogeneity was considered when the I^2^ value was greater than 50% and the p-value was less than 0.05. The sources of heterogeneity between studies were not explored.

### Reporting bias assessment

As recommended by the Cochrane methodology [21], funnel plots followed by the Egger’s test were used to assess publication bias for the outcome ‘‘mortality” using MedCalc version 22.019. This was performed only when sufficient data were available (>10 studies). Certainty was not assessed.

## Results and discussion

### Number of eligible studies

**Fig 1:**
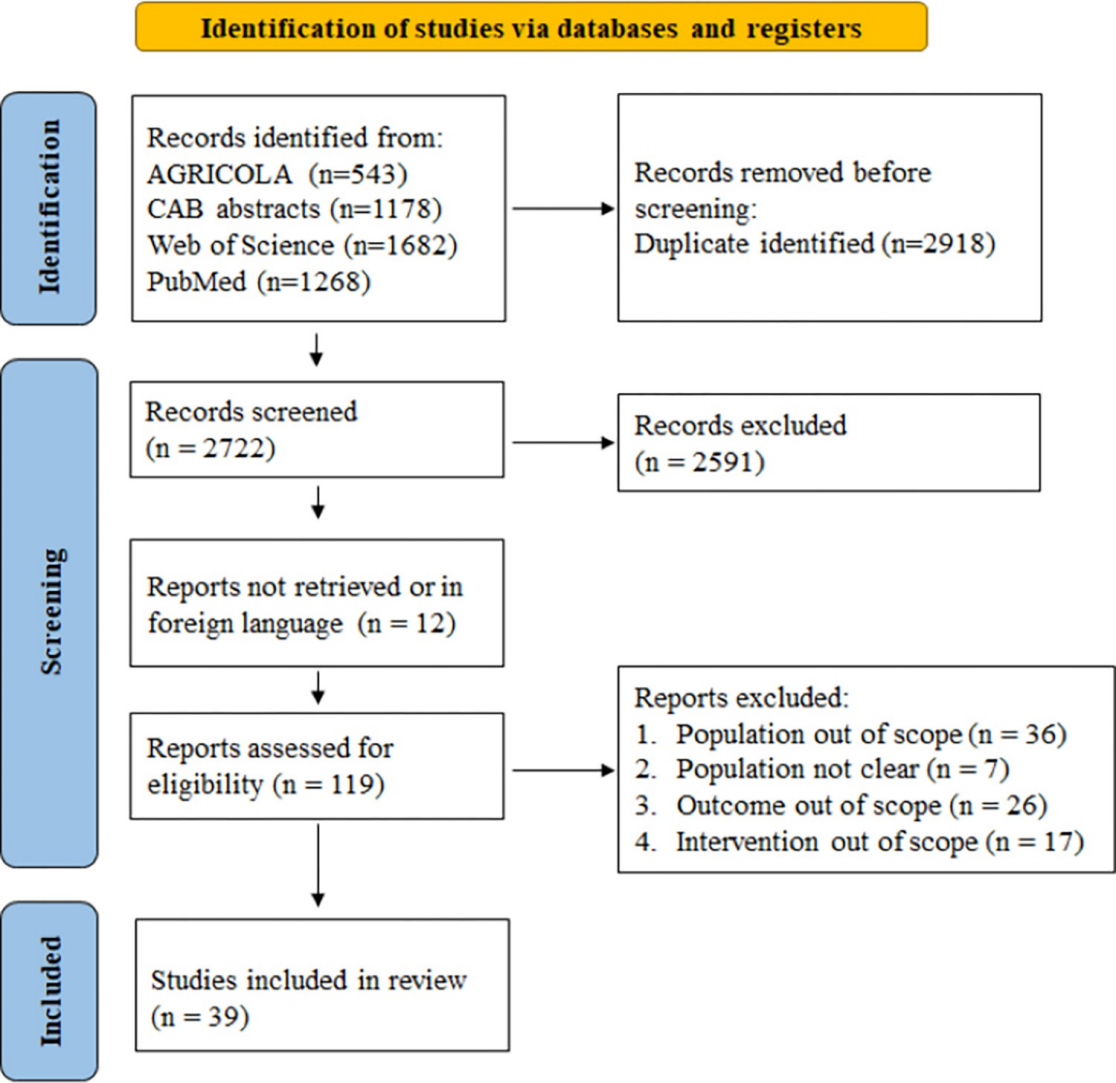
Methodological PRISMA (Preferred Reporting Items for Systematic Review and Meta-analysis) flowchart for the selection of studies.

The results of the selected studies, based on the inclusion criteria, are summarized in Fig 1. Initially, 2,722 studies were identified from the selected databases. After removing non-eligible studies based on the inclusion criteria, 39 studies were deemed suitable for data extraction.

### Studies characteristics

**Fig 2:**
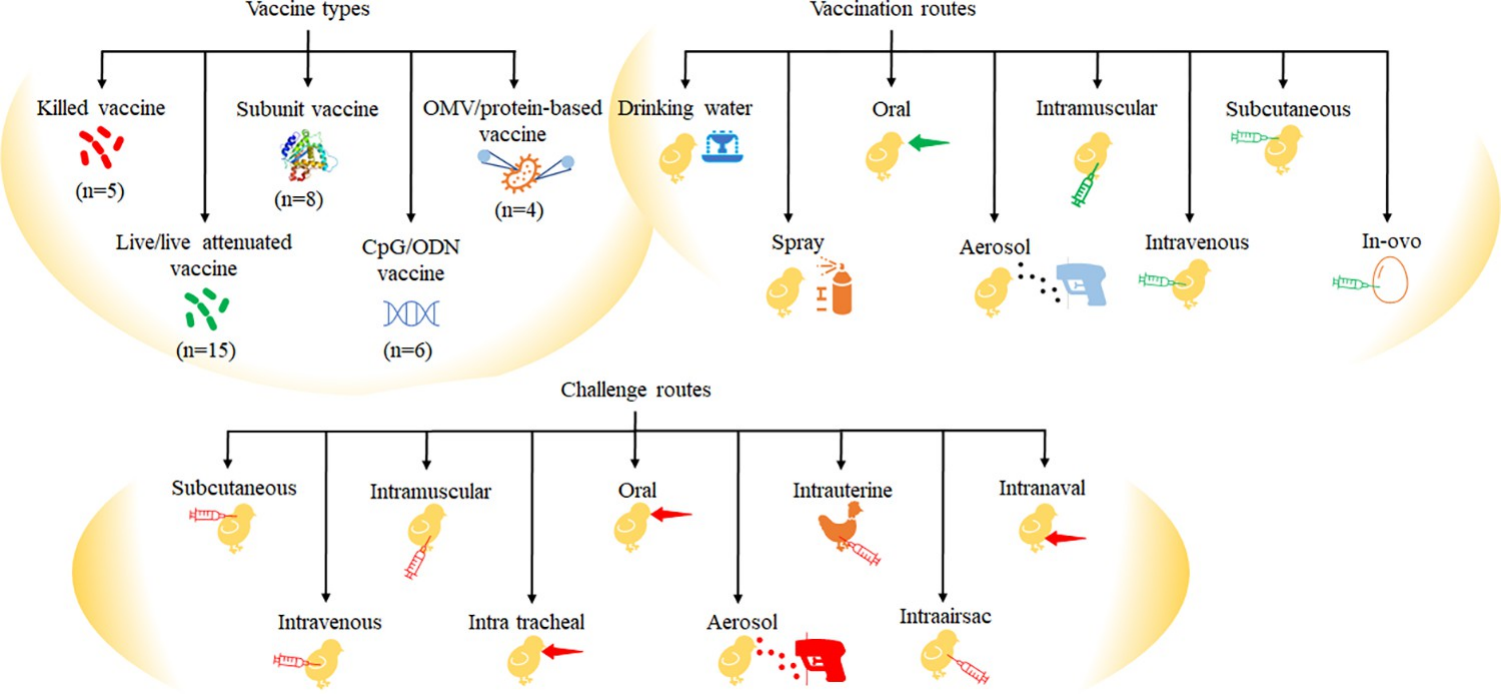
*E*. *coli* vaccine types, vaccination routes and challenge routes used to assess vaccination efficacy in broilers. “n" refers to number of studies.

In total, 39 studies were selected for data extraction. Various types of *E*. *coli* vaccines with different efficacy were reported in the selected studies (Fig 2), including killed (n = 5), subunit (n = 8), outer membrane vesicles/protein based (n = 4), live/live-attenuated (n = 16) and CpG-ODN (n = 6) vaccines. Ten studies evaluated the efficacy of a commercially available vaccine, while others aimed to assess the suitability of newly reported vaccine candidates. Mortality was considered as an assessment parameter in all studies. Additionally, FCR was included in most studies, except for five.

### Vaccine types

Characteristics of *E*. *coli* vaccine types in broiler chickens are described below.

### Killed vaccines

Vaccines using inactivated bacteria can elicit an immune response, mainly through humoral immunity. These vaccines are considered safe as they do not replicate within the host. Hyperimmunization of chickens with intravenous injection of a heat-killed *E*. *coli* J5 strain was shown to be effective in preventing mortality and pathological lesions following a challenge [27] (Table 2, S1 Table). In another study formalin treatment, irradiation and ultrasonication methods were employed for bacterial inactivation, all of which were shown effective in preventing lesions [28]. Sayed et al. (2021) also showed that formalin-killed *E*. *coli* vaccination significantly reduced mortality after challenge and could be combined with an avian influenza vaccine prepared using the same method, which can also induce a higher antibody response in birds [29]. Recently, autogenous vaccines have been used as a potential solution to address the heterogeneity of *E*. *coli* isolates. However, there is limited evidence regarding their effectiveness. In a study conducted by Keita et al. (2022), passive immunization using a bivalent formalin-killed autogenous vaccine was found to be effective in reducing mortality when chicks were challenged with one of two *E*. *coli* strains [30]. This indicates strain-specific protection provided by the vaccine. Additionally, a combination approach involving parent stock vaccination with an autogenous vaccine, along with the supplementation of feed with *Enterococcus faecium* DSM 7134 and fructo-oligosaccharides in the progeny, showed benefits in terms of improving body weight and gut health. However, FCR was not affected by this combination approach [31]. All of the above-mentioned vaccines have not yet reached to the commercial market.

**Table 2:** Important characteristics of killed vaccines against colibacillosis in broiler chickens.

Reference	Day of vaccination	Route of vaccination	Dose and route of challenge	Important findings
Abdul-Aziz & El-Sukhon, 1998 [27]	5, 14 & 20	IV	0.2 ml of 6x10^8^ CFU/ml (IV)	Chickens hyperimmunized with *E*. *coli* J5 showed protection based on mortality, clinical signs and pathological lesions.
Ibrahim et al., 1997 [28]	14	IM	10^8^ CFU/bird	Formalin-killed, irradiated and ultrasonicated *E*. *coli* induced protection.
Sayed et al., 2021 [29]	21 & 42	SC	0.2 ml of 10^7^ CFU (IM)	Formalin-killed *E*. *coli* prevented mortality from experimental *E*. *coli* infection and can be combined with inactivated avian influenza vaccine.
Keita et al., 2022 [30]	20 & 22 weeks	IM^1^	0.1 ml of 3x10^8^ CFU/ml (SC)^2^	Breeders received a bivalent autogenous vaccine; passive immunization was effective in chicks against challenges with one out two *E*. *coli* stains only
Fuhrmann et al., 2022 [31]	12 & 17 weeks	IM^1^	0.6 ml of 3.2x10^7^ CFU/ml (oral)^2^	Passive immunization together with administration of pre-and probiotics have beneficial effects on body weight and gut health.

IV: intravenous, IM: intramuscular, SC: subcutaneous, CFU: colony forming unit; ^1^vaccination of breeders for passive immunization; ^2^ challenge of progenies

### Subunit vaccines

These vaccines consist of purified antigenic parts, such as proteins or protein fragments, rather than the entire pathogen [32]. Table 3 provides a summary of the key characteristics of subunit *E*. *coli* vaccines in broiler chickens. Details of each study are provided in S1 Table. Vandemaele et al. (2006) investigated the impact of immunization with the biologically active lectin domain of PapGII on the avian immune response to APEC O78 challenge [33]. The results showed that while immunization effectively stimulated the production of adhesion-inhibiting antibodies, it did not provide protection against APEC O78 infection through coarse spray or intra-air sac challenge. Similar results were observed when the sugar-binding domain of FimH (FimH156) was used [34]. However, Lynne et al. (2012) demonstrated that vaccinating birds with increased serum survival gene (*iss*) fusion proteins provoked the serum and mucosal antibody response and consequently resulted in broad protection against bacterial challenges with three different APEC strains: O1, O2 and O78 [35]. Similarly, a modified strain of *Salmonella* carrying multiple genes from *E*. *coli*, including P-fimbriae (*papA*, *papG*), aerobactin receptor (*iutA*) and CS31A surface antigen (*clpG*), elicited mucosal and systemic antibody responses, and stimulated lymphocytic proliferation [36]. Although a single vaccination with this attenuated *Salmonella* strain only provided partial protection against *E*. *coli* challenge, repeated vaccination significantly enhanced the protective response. Co-administration of this vaccine candidate with a live attenuated *Salmonella* expressing the heat-labile toxin of *E*. *coli* B subunit (LTB) as an adjuvant proved to be more effective in reducing mortality and morbidity rates in challenged birds [37]. In a separate study, a probiotic bacterium called *Lactobacillus saerimneri* was used as a delivery system to create a recombinant vaccine that expressed fimbrial subunit A (FimA) and outer membrane protein C (OmpC) of O78 APEC. Oral administration of the recombinant *L*. *saerimneri* effectively induced an antigen-specific immune response and provided protection, as 70% of vaccinated birds survived while 100% mortality was observed in the non-vaccinated challenge control group [38]. Additionally, a non-adjuvanted liposome-encapsulated mixture of rough LPSs exhibited a positive dose-dependent effect, especially in terms of antibody level in birds. Immunization with the highest dose (5μg) resulted in lower lesion scores and increased body weight, although the mortality rate did not show a significant difference [39].

**Table 3:** Important characteristics of subunit vaccines against colibacillosis in broiler chickens.

Reference	Day of vaccination	Route of vaccination	Dose and route of challenge	Important findings
Vandemaele et al., 2005 [34]	10 (single) or 10 & 30 (with booster)	IM or IN	10 ml/group, 3x10^10^ CFU/ml; prior infection with NDV (nebulization)	Immunization with sugar-binding domain of FimH (FimH156) effectively induced high levels of adhesion-inhibiting antibodies but did not provide protection against APEC O78 infection.
Vandemaele et al., 2006 [33]	10	IM	10 ml/group, 2.7x10^10^ CFU/ml (prior infection with NDV; nebulization) or 0.2 ml 10^4^ (intra-airsac)	Immunization with the biologically active lectin domain of PapGII could effectively induce high levels of adhesion-inhibiting antibodies but did not provide protection against APEC O78 infection delivered via coarse spray or via intra-air sac challenge.
Lynne et al., 2012 [35]	14	IM	0.1 ml of 10^7^ CFU (intra-airsac)	The Iss antigen provided significant protection against challenges with three different APEC strains (O1, O2 and O78).
Chaudhari et al., 2013 [36]	1 (single) or 1 & 14 (with booster)	Oral	50 μl of 10^7^ CFU (intra-airsac)	Prime and boost vaccination with an attenuated *Salmonella* strain carrying P-fimbriae (*papA*, *papG*), aerobactin receptor (*iutA*) and CS31A surface antigen (*clpG*) genes of *E*. *coli* induced immune response and provided protection against *E*. *coli* challenge.
Chaudhari & Lee, 2013 [37]	1	Oral	0.1 ml of 10^6^ CFU (intra-airsac)	Coadministration of live attenuated *Salmonella* strain expressing the heat-liable toxin of *E*. *coli* B subunit (LTB) increased the efficacy of the *Salmonella*-delivered APEC vaccine developed by Chaudhari et al. (2013).
Ma et al., 2018 [38]	1–3 & 14–16	Oral	5x10^11^ CFU (Oral)	Immunization of birds with a recombinant *Lactobacillus saerimneri* expressing FimA and OmpC antigen of O78 APEC provided protection.
Dissanayake et al., 2010 [39]	7 & 21	IM	10^6^ CFU (subcutaneous)	Liposome-encapsulated mixture of rough LPSs significantly lowered lesion scores and increased body weight but no difference was observed in mortality.
Tuntufye et al., 2012 [40]	10 & 24	Intranasally & IM	2x10^6^ CFU (intra airsac)	Four ferri-siderophore receptors (FuhE, FepA, IroN, IutA) were expressed in live or bacterial ghost cells; none of the two recombinants were protective.

IM: intramuscular, IN: intra nasal, CFU: colony forming unit

Iron uptake system genes play a crucial role in the virulence mechanism of APEC. Four ferri-siderophore receptors namely FuhE, FepA, IroN, IutA were expressed in recombinant live or bacterial ghost cells [40]. However, despite increased IgG titers in birds, neither the intranasal administration of recombinant live *E*. *coli* nor the intramuscular infection of recombinant ghost cells was able to reduce mortality and lesion scores, leading to the conclusion that both vaccine candidates were non-protective.

### Outer membrane vesicles/proteins-based vaccines

Outer membrane vesicles (OMVs) are naturally derived spherical nanovesicles originating from the bacterial outer membrane which contains various bacterial components, such as lipopolysaccharides, proteins, and other antigens [41]. Their protective efficacy was evaluated in broiler chickens as shown in Table 4 and S1 Table. Immunization of birds with a nanosized OMV-based vaccine derived from APEC O2 demonstrated no adverse effects. Moreover, it significantly increased the survival rate, reduced bacterial loads, and suppressed the production of proinflammatory cytokines [42]. Immunologically, the vaccine primarily stimulated antigen-specific antibody responses and IFN-γ mediated immune responses in the host. Taking a step further, a combination of multi-serogroup OMVs derived from O1, O2 and O78 *E*. *coli* strains induced a robust non-specific and antigen specific immune responses. This was evident from the production of IgG antibodies specific to APEC antigens and resulted in a 90–100% increase in protection against challenges with APEC O1, O2 or O78 strains compared to the control group [43]. It is difficult to attribute the observed protection solely to specific proteins or polysaccharides within the OMVs due to their complex composition.

**Table 4:** Important characteristics of outer membrane vesicles/protein-based vaccines against colibacillosis in broiler chickens.

Reference	Day of vaccination	Route of vaccination	Dose and route of challenge	Important findings
Hu et al., 2020 [43]	7 & 14	IM	5x10^8^ CFU (intra-airsac)	Vaccination with nanosized OMVs had no side effects and efficiently protected chicks against homologous infection with APEC O2. It provoked antibody and IFN-γ mediated immune responses.
Hu et al., 2020 [42]	7 & 14 & 21	IM	5x10^8^ CFU (intra-tracheal)	Combined OMVs from O1, O2 and O78 strains provided robust and broad protection against *E*. *coli* challenges with all three strains.
Mohammed et al., 2020 [46]	21	SC	10^7^ CFU (IM)	Addition of chitosan and ascorbate chitosan nanoparticles improved the immune response induced by outer membrane proteins and flagellin.
Abd El-Aziz et al., 2022 [47]	14	SC	10^7^ CFU (IM)	Chitosan loaded nanoparticles with Montanide adjuvant enhanced immunity for a longer time period.

IM: intramuscular, SC: sub cutaneous, CFU: colony forming unit

To enhance vaccine uptake and improve pharmacokinetic and pharmacodynamic properties, nanotechnology-based vaccines are beneficial. These vaccines are designed to boost the immune response by providing antigenic targets in a way that mimics natural infection, improving stability and targeting specific immune cells [44, 45]. Mohammed et al. (2021) investigated the potential of chitosan nanoparticles in enhancing the immune response of chickens after vaccination with the outer membrane proteins (OMPs) and flagellar antigens from O1 and O78 serogroups [46]. The study utilized two types of chitosan nanoparticles, namely the characterized chitosan (CS) and ascorbate chitosan (AsCS), in both loaded and encapsulated forms. The results demonstrated that both forms of chitosan nanoparticles improved the immune response, in terms of antibody production in chickens and provided protection against infections induced by *E*. *coli* O1 and O78, compared to the control group. Subsequent research by Abd El-Aziz et al. (2022) corroborated these findings and further demonstrated that the addition of Montanide as an adjuvant to the chitosan nanoparticles prolonged the humoral and cell-mediated immunological responses, thereby enhancing immunity for an extended period [47].

### Live and live attenuated vaccines

Live and live attenuated vaccines have advantages for mass application, as they can be delivered as spray or via drinking water. Most of the studies in this review evaluated the efficacy of these vaccines (Table 5, S1 Table). A study by Frommer et al. (1994) found that a non-pathogenic piliated *E*. *coli* strain provided broad protection against colibacillosis-induced mortality caused by O1:K1, O2:K1 and O78:K80 [48]. Immunization at 14 or 21 days of age was more effective than at an early age (1 or 7 days) and drinking water or intramuscular administration showed better efficacy than the spray method. Kariyawasam et al. (2002) demonstrated that administering a live *E*. *coli* strain of O78 serotype via aerosol route at 18 days of age reduced pathological lesions and systemic bacterial colonization when challenged with the same strain [49].

**Table 5:** Important characteristics of live and live attenuated vaccines against colibacillosis in broiler chickens.

Reference	Day of vaccination	Route of vaccination	Dose and route of challenge	Important findings
***Live vaccine***				
Frommer et al., 1994 [48]	1, 7, 14 or 21	IM, spray or per os	1x10^8^ CFU (IM)	Vaccination at the age of 14 or 21, but not at 1 or 7 days, via IM or per os elicited protection; mortality was reduced after challenge with O1, O2 and O78 *E*. *coli* challenges; spray vaccination provided inadequate protection.
Kariyawasam et al., 2002 [49]	18	Aerosol	10^8^ CFU (Intra airsac); together with IBV	Vaccination reduced pathological lesions and systemic bacterial colonization after homologous challenge.
***Live attenuated vaccine***				
Peighambari et al., 2002 [50]	14 or 10 and 14	Coase spray	0.1x10^9^ CFU (prior infection with IBV)	Double mutant was created deleting *cya* and *crp* genes of O2 and o78 strains; moderate protection was observed with mutant O2 strain as it reduced air sac lesions but the mutant O78 was not effective.
Kariyawasam et al., 2004 [51]	1 and 14	Coarse spray	100 ml/group of 10^9^ CFU/ml (aerosol)	Mutants of *gal*E, *pur*A or *aro*A from O78 strain provided homologous but not heterologous (O2) protection.
Asaad et al., 2019 [52]	1	Fine spray (crp-deletion mutant)	6x10^8^ CFU (IT)	Both vaccines were effective to minimize the pathological lesions following homo-and heterologous (O1) challenges.
		Eye drop (aroA deletion mutant)		
Abd El-Mawgoud et al., 2020 [53]	1	Fine spray	0.5 ml of 10^8^ CFU/ml (SC)	The *crp* deletion mutant vaccine was efficacious to reduce mortality and bacterial colonization after homologous challenge but was not effective against heterologous challenge (O125).
Sadeghi et al., 2018 [54]	1	Coarse spray	10^8^ (IT)	*aro*A deletion mutant (Poulvac) reduced clinical signs and lesions due to O78 and untypeable *E*. *coli* strains; no difference was observed in mortality.
Galal et al., 2018 [55]	1	Coarse spray	10^9^ CFU (IT)	*aro*A deletion mutant (Poulvac) provided protection against the homologous challenge; the vaccine did not interfere with humoral immune response induced by other vaccines such as AI, NDV, IBV or IBD.
Rawiwet et al., 2009 [59]	5	Oral	0.5 ml of 1.2x10^9^ CFU/ml (IT)	*aro*A deletion mutant (Poulvac) reduced morbidity (pathological lesions) but no difference was seen following homologous challenge.
Mohammed et al., 2016 [56]	1	Coarse spray or drinking water	6x10^8^ CFU (IT)	Spray vaccination of *aro*A deletion mutant (Poulvac) led to significant reduction in pathological lesions but drinking water application was not effective; homologous protection was observed but not the heterologous protection against O1 challenge
Gharib et al., 2017 [57]	1 or 1 and 14	Coarse spray	9x10^8^ CFU (IT)	*aro*A deletion mutant (Poulvac) was effective against O78 but not against O125 challenge, protection was associated with cell mediated immunity.
Elbestawy et al., 2021 [60]	1	Coarse spray	0.5 ml of 1.2x10^8^ CFU (IT)	*aro*A deletion mutant (Poulvac) provided protection against O27 and O8 assessed with FCR, mortality, lesions, clinical signs and bacterial re-isolation; protection against O115 was not significant.
Tarabees et al., 2019 [58]	1 and 15	Coarse spray	0.5 ml of 1x10^8^ CFU (oral)	*aro*A deletion mutant (Poulvac) decreased the mortality rate and bacterial colonization after homologous challenge; vaccine response was improved by supplementation of probiotics *Enterococcus faecalis*.
Galal et al., 2021 [61]	1 or 7	Coarse spray	0.1 ml of 10^9^ CFU/ml (IT)	*aro*A deletion mutant (Poulvac) given at 7 days in birds with prior treatment with lincospectin 100 for 3 days was the most effective to prevent mortality and loss of performance due to challenge with O78 strain.
Li et al., 2017 [63]	1 day and 12 weeks	Coarse spray	0.1 ml of 5x10^6^ CFU/ml (intrauterine)	*aro*A deletion mutant (Poulvac) alone was not protective; Poulvac followed by autogenous vaccine delayed the onset of clinical signs for 3–4 days but no signs of protection against homo-and heterologous challenges.
Šenk et al., 2022 [73]	Poulvac (12 & 20 weeks) with/without autogenous vaccine (18 weeks)	Poulvac: Spray	ND	Vaccinating birds with both commercial live-attenuated (Poulvac) and autogenous vaccines showed some benefits compared to using only the live-attenuated vaccine.
		Autogenous: IM		

IM: intramuscular, CFU: colony forming unit, IT: intra tracheal, ND: not done

Advancements in understanding genetic characteristics of *E*. *coli* led to the development of live attenuated vaccine candidates by targeting essential genes required for multiplication in the host. Peighambari et al. (2002) tested the efficacy of double mutants created by deletion of *cya* and *crp* genes in O2 and O78 *E*. *coli* strains [50]. The mutant O2 strain provided moderate protection against air sac lesions when administered via spray, while the mutant O78 strain was ineffective. It was also observed that antibody response was not stimulated in vaccinated birds, indicating the importance of innate or adaptive immunity for protection against colibacillosis. Other mutants with *gal*E, *pur*A or *aro*A deletions showed similar immunogenicity and serogroup-specific protection but they did not provide effective cross-protection against heterologous challenge [51].

Currently, there are two licensed live attenuated vaccines against *E*. *coli* infection. The *crp* deletion mutant of *E*. *coli-*O78 (Nisseiken Co., Ltd., Tokyo, Japan) is marketed in Japan and is recommended to be administered via fine spray (particle size <20 μm) in day-old chickens. The vaccine has shown effectiveness in reducing lesions following the challenge with homo- and heterologous strains [52, 53], although it may be ineffective against heterologous challenge based on mortality pattern, clinical signs, pathology and bacterial re-isolation [53]. The second live attenuated vaccine is developed by deleting *aro*A gene of *E*. *coli*-O78 strain (Poulvac®, E. coli, Zoetis), and is usually administered via coarse spray in day-old chicks. In a field trial, the Poulvac vaccine did not affect the weight gain in broiler chickens [54]. In experimental conditions, several studies have suggested its efficacy against homologous challenge in reducing colibacillosis-associated pathological lesions [54–58]. Oral vaccination of birds at day 5 of age reduced morbidity [59] but drinking water application was found to be ineffective in inducing protection [56]. The findings regarding heterologous protection were not consistent among studies with some showing effectiveness against certain strains [54, 60], but not others [56, 57, 60]. The efficacy of the vaccine against homologous challenge was enhanced by supplementation of probiotics *Enterococcus faecalis* [58] and pre-treatment with Lincospectin improved the vaccine’s response [61]. However, the immune response elicited by the live attenuated vaccine was reported to be interfered by the prior application of ceftiofur sodium antibiotic in layer birds [62]. Recently, Li et al. (2017) reported that Poulvac administered alone at 1 and 12 weeks did not protect birds against intrauterine challenge despite high antibody titers [63]. However, when the live attenuated vaccine was followed by an autogenous vaccine, the onset of disease was delayed, but there was no evidence of protection against homologous or heterologous challenges.

### CpG-ODN vaccines

CpG oligodeoxynucleotides (CpG ODN) are synthetic DNA molecules that contain specific patterns of cytosine and guanine bases (CpG) recognized by the immune system. They have been used as vaccine adjuvants to enhance the immune response to antigens, including viral or bacterial protein [64]. Several studies investigated the effectiveness of CpG ODN vaccines in reducing mortality caused by colibacillosis (Table 6, S1 Table). Gunawardana et al. (2019) explored the use of CpG ODN administered *in ovo* to stimulate the immune system of newly hatched chicks and protect them against subcutaneous (SC) bacterial challenge with *E*. *coli* serogroup O2 [65]. The study revealed that the administration of synthetic CpG-ODN in freshly hatched chicks led to a rapid increase in immune cells, such as macrophages and dendritic cells, as well as cytokine responses in spleen and lungs. The authors also observed enhanced protection against bacterial challenge in the chickens treated with synthetic CpG-ODN, as indicated by reduced bacterial loads in various tissues and increased survival rates. Similar findings were reported in the studies conducted by Taghavi et al. (2009) and Gomis et al. (2004), which employed similar study designs involving *in ovo* vaccination, SC challenge with *E*. *coli* O2 at comparable doses, and similar observation period for mortality [66, 67]. Two additional studies examined the effects of CpG-ODN vaccines in newly hatched chicks but with different study designs. Allan et al. (2018) also employed *in ovo* delivery, however the challenge with *E*. *coli* O2 was done via the intranavel route and at a much lower dosage (25 CFUs vs 10^5^ CFUs in the aforementioned studies) [68]. Nevertheless, the authors observed increased survival rates in chicks compared to the control group. In a study by Sarfraz et al. (2022) using a similar vaccination route and challenge model, the effectiveness of different innate immune stimulants and their combination was compared [69]. The results showed that the *in ovo* administration of CpG-ODN in conjunction with polyinosinic-polycytidylic acid was the most efficient in protecting chicks when they were challenged via intranasal route. In another study, one-day-old chicks received intrapulmonary delivery of CpG-ODN followed by SC challenge with *E*. *coli* O2, resulting in approximately half the relative risk of mortality compared to birds that received saline [70].

**Table 6:** Important characteristics of CpG-ODN vaccines against colibacillosis in broiler chickens.

Reference	Day of vaccination	Route of vaccination	Dose and route of challenge	Important findings
**CpG-ODN**				
Gomis et al., 2004 [67]	in ovo	in ovo	10^5^ CFU (SC)	Chickens treated with synthetic CpG-ODN showed enhanced protection against bacterial challenge, indicated by reduced bacterial loads in various tissues and increased survival rates.
Taghavi et al., 2009 [66]	in ovo	in ovo	10^4.5^ CFU (SC)	Chickens treated with synthetic CpG-ODN showed enhanced protection against bacterial challenge, indicated by reduced bacterial loads in various tissues and increased survival rates.
Goonewardene et al., 2017 [70]	1	intrapulmonary	10^4.5^ CFU (SC)	SC challenge with E. coli O2, resulting in about half of the relative risk of mortality as did the birds that received saline
Allan et al., 2018 [68]	in ovo	in ovo	25 CFU (intranavel)	Challenge with E. coli O2 was done via the intranavel route and at a much lower dosage; increased survival rates of chicks in their experiments compared to the control group.
Gunawardana et al., 2019 [65]	in ovo	in ovo	10^4.5^ CFU (SC)	In rapid increase of immune cells such as macrophages and dendritic cells; enhanced protection against bacterial challenge, indicated by reduced bacterial loads in various tissues and increased survival rates.
Sarfraz et al., 2022 [69]	in ovo	in ovo	25–30 CFU (intranavel)	Coadministration of CpG (10μg/embryo) and poly I:C 15μg/embryo provided 100% protection against experimental yolk sac infection.

SC: subcutaneous, CFU: colony forming unit

### Field studies

Only a limited number of studies (Table 7, S1 Table) examined the efficacy of *E*. *coli* vaccines in field settings compared to the experimental studies mentioned above. The first two studies [71, 72] were not considered for data extraction, as they did not meet the selection criteria. One of them evaluated the effectiveness of a commercially available inactivated subunit vaccine, which contains *E*. *coli* fimbrial antigen and flagellar toxin (Nobilis®, MSD Animal Health). The vaccine was administered intramuscularly to commercial broiler breeders [71]. The results showed that the vaccinated flocks experienced lower mortality potentially associated with natural *E*. *coli* infection. However, there were no significant differences observed in terms of first week mortality in chicks, slaughterhouse condemnation rates and FCR between birds from vaccinated or non-vaccinated breeder flocks. Another study investigated the efficacy of the live attenuated Poulvac® E. coli vaccine, which was administered in day old chicks as recommended [72]. The findings demonstrated that colibacillosis-like lesions were less frequent in vaccinated flocks compared to non-vaccinated flocks. However, no differences were observed in FCR between the two groups. Another study showed that colibacillosis-related lesions were observed less frequently in the flock of birds vaccinated with both live attenuated Poulvac® and autogenous vaccines compared to the group vaccinated only with live attenuated vaccine, indicating some benefits of combining both vaccines in the field [73].

**Table 7:** Important characteristics of field studies vaccination against colibacillosis in broiler chickens.

Reference	Age of vaccination	Route of vaccination	Dose and route of challenge	Important findings
Gregersen et al., 2010 [71]	12 & 18 weeks	IM	ND	Vaccinated breeder flock experienced less mortality due to E. coli natural infection but the vaccination no beneficial impact on the first week mortality of chicks.
Mombarg et al., 2014 [72]	day old	Spray	ND	Colibacillosis associated lesions recorded in slaughterhouse were less frequent in vaccinated flocks compared to non-vaccinated flocks.

IM: intramuscular, ND: not done

### Risk of bias

**Fig 3:**
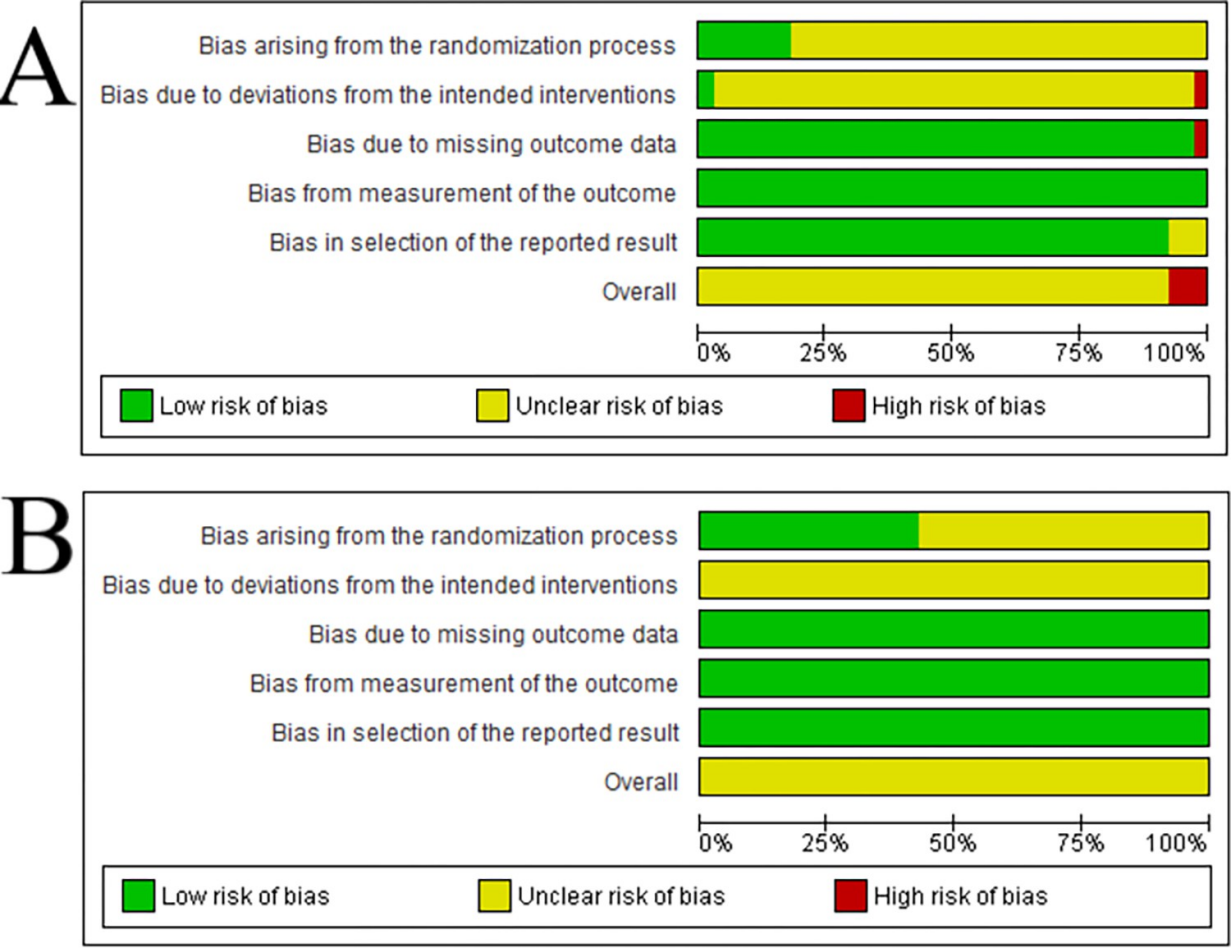
Risk of Bias (RoB) in the *E*. *coli* vaccination studies that reported mortality (A) or feed conversion ratio (B) as one of the assessment parameters.

In the evaluation of 39 papers reporting mortality data, the overall RoB revealed ‘‘some concerns” for the majority (n = 36, 92.3%) and ‘‘high risk” for a small fraction (n = 3, 7.7%) (Fig 3A). When examining seven studies that included FCR data, the overall RoB was evaluated as ‘‘unclear’ (Fig 3B). For mortality outcome, one study (2.6%) exhibited “high risk” of bias, one (2.6%) ‘‘low risk”, and 37 (94.8%) were assessed to have ‘‘unclear” (Fig 3B). The overall risk of bias of domains 3 to 5 was assessed as ‘‘low” for FCR. In both mortality and FCR, domains 1 and 2 emerged as the primary sources of bias. The overall bias arising from the randomization process (domain 1) recorded is mainly related to the lack of information concerning concealed allocation sequence of animals in the groups. For example, for mortality as outcome, only seven studies (n = 17.9%) provided this information. The concern with the result of the domain 2 (bias due to deviation from the intended intervention) is due to the absence of information concerning the awareness or not of animal caregivers/researchers about the assigned interventions and whether there were deviations from the intended intervention that arose because of the trial. Again, only one study (n = 2.6%) provided these details in the papers with mortality as outcome.

### Meta-analysis

**Fig 4:**
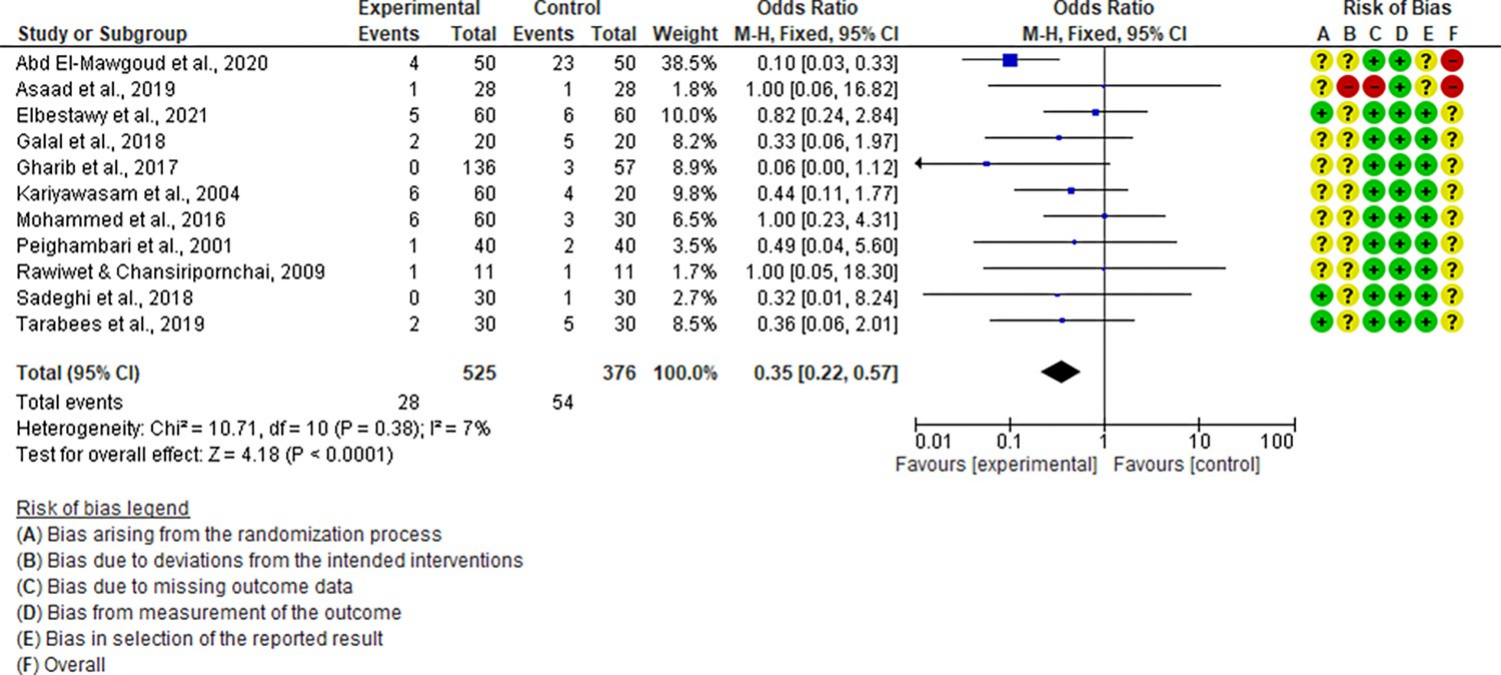
Forest plot of live attenuated vaccine efficacy considering the “mortality” assessment parameter.

**Fig 5:**
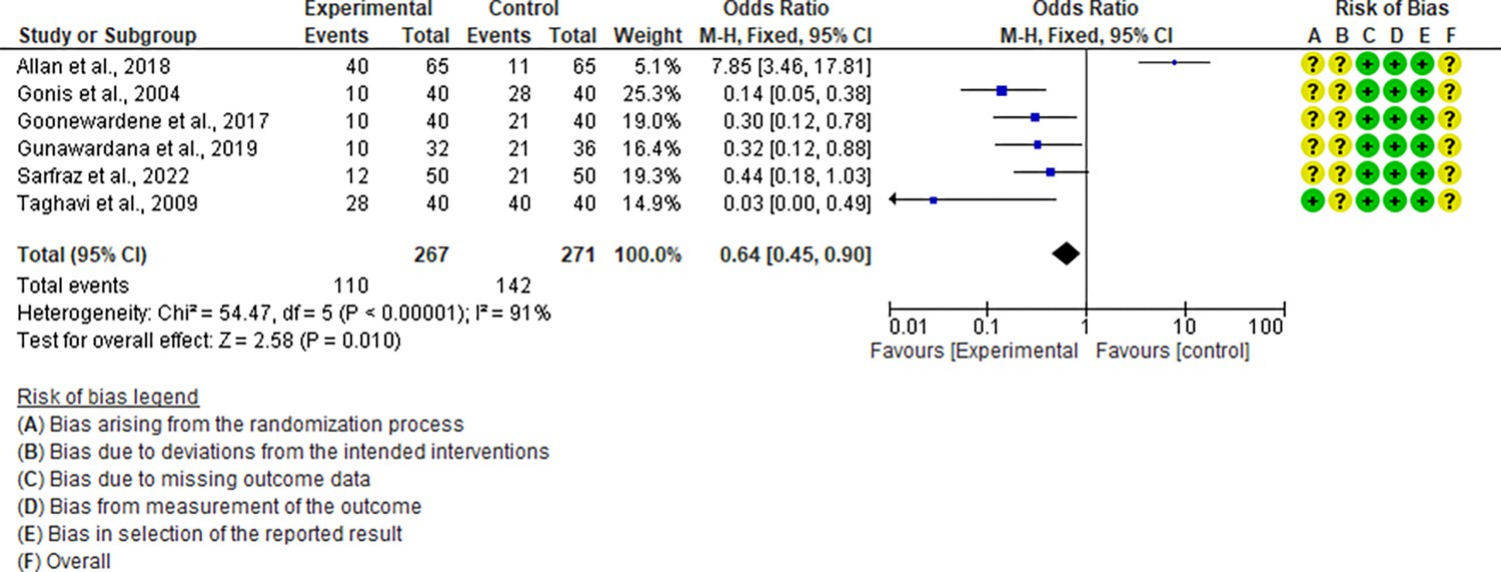
Forest plot of CpG oligodeoxynucleotides (CpG-ODN) vaccine efficacy considering the “mortality” assessment parameter.

For the live attenuated vaccine, a total of twelve studies were included in the meta-analysis (Fig 4). All studies were performed after the year 2000 and the majority (83.3%) had an overall RoB assessed as “unclear”. When considering the effects on mortality, the comparison between the non-vaccinated and vaccinated groups showed a significant (P < 0.00001) trend favoring vaccination with a pooled odds ratio of 0.30 (95% CI: 0.19–0.48). A minimal but non-significant level of heterogeneity (P = 0.21; I^2^ = 24%) among studies was recorded. Due to the absence of a significant heterogeneity among included studies, the subgroup and meta-regression analysis were not performed. As presented in Fig 5, the six studies included in the meta-analysis of the CpG-ODN vaccine had an overall RoB assessed as ‘unclear’ and a high level of heterogeneity (P < 0.00001; I^2^ = 91%). The pooled odds ratio for mortality was 0.64 (95% CI: 0.45–0.90). Due to the limited number of studies included, the sources of heterogeneity were not assessed.

**Fig 6:**
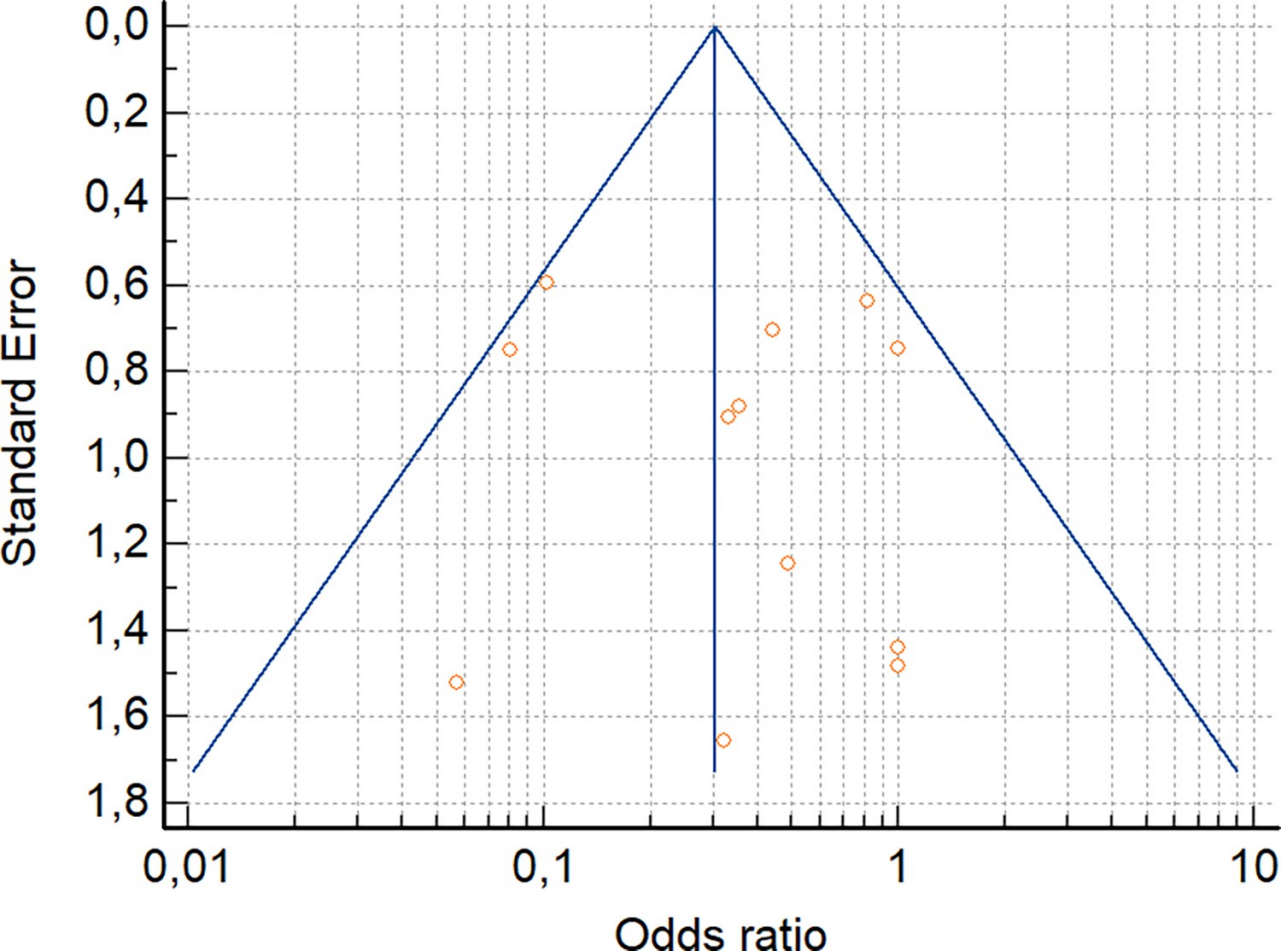
Funnel plot of live attenuated vaccine efficacy considering the “mortality” assessment parameter.

The reporting bias was assessed only for the live attenuated vaccine efficacy considering the “mortality” as outcome because more than ten studies were included in the meta-analysis. The funnel plot (Fig 6) and the Egger’s test results (Intercept = 0.43, 95% CI [-1.94 to 2.80], P = 0.69) showed a symmetry of the studies and a non-significant regression test, respectively, indicating an absence of publication bias (studies with no significant results) and validating the analysis as reasonable and reliable.

### Outlook and conclusion

Colibacillosis in broiler chickens poses challenges for animal health and welfare, with serious economic consequences, impacting food safety and security, which can have a clear effect on consumers’ wellbeing and livelihoods. This systematic review examines the efficacy of various vaccines in preventing colibacillosis in broilers, revealing that, while some vaccine candidates have shown promising results, challenges and limitations remain that need to be addressed.

Killed and subunit vaccines, while being safe due to only exposing animals to fragments of the pathogen, have limited range of protection and require injections in birds. This ultimately raises concerns about the practicality and cost-effectiveness in commercial broiler production. On the other hand, even though live attenuated vaccines can be mass-applied through practical routes, such as drinking water or spray, rigorous safety assessments and investigations into potential long-term effects are still necessary.

In this study, meta-analysis was possible only for live-attenuated and CpG ODN vaccines, reflecting great heterogenicity among studies. While the analysis demonstrated a significant trend favoring these vaccination types in reducing mortality, there is a significant variation in challenge models used in vaccine research for *E*. *coli* infections, involving various routes of infection, predisposing factors, and a wide range of challenge doses. It shows the difficulty in reproducing the disease experimentally. Nevertheless, standardizing the challenge model is essential for consistent evaluation of vaccine candidates and comparison between studies [74]. Therefore, expanding the portfolio of *E*. *coli* vaccines, considering practical feasibility and serotype-independent protection, as well as establishing a robust infection model, are crucial. Field studies offer insights into the real-world vaccine effectiveness, yet the limited number of studies found in this systematic review highlights the need for more research, namely to evaluate vaccine efficacy in field conditions and assess additional parameters such as pathological consequences, economic impact and long-term protection.

Developing an effective vaccine against colibacillosis in chickens is complex due to the high heterogenicity of *E*. *coli* isolates, elusive disease mechanisms, and absence of definitive markers for pathogenic isolates [17, 20]. This complexity is evident in the limited number of vaccines reaching the commercial market, with conflicting reports about their effectiveness [53, 60].

Future vaccine development requires a multi-dimensional approach, focusing on identifying conserved antigens that confer broad protection across different APEC serotypes or incorporate antigens that confer broad protection across different APEC serogroups. Multivalent vaccines targeting multiple serogroups or incorporating diverse antigens may offer enhanced efficacy and broader coverage. Exploring innovative technology, such as irradiation [75] or glycoconjugate vaccines [76], may hold promise for improving vaccine delivery and bird protection against colibacillosis. Increased investment in research and development along with collaborative efforts between academic, industry and regulatory agencies, can expedite the translation of promising vaccine candidates into commercial products. Public-private partnerships and funding initiative can also incentivize vaccine development for diseases, such as colibacillosis, with significant impacts on animal health and economic sustainability. Despite providing valuable insights, this review has limitations. The focus was primarily on broiler production chain, excluding vaccine types and studies related to the *E*. *coli* vaccination in layer birds. Subgroup analysis was challenging due to variations in challenge models and experimental designs. The meta-analysis results should be interpreted with caution considering the diversity of influencing factors and the reduced number of studies considered. Additionally, studies that did not include mortality, FCR and condemnation at slaughter as assessment criteria were excluded, although reproducing colibacillosis in experimentally infected birds is challenging, leading to exclusion of some vaccination studies in broilers.

In conclusion, while significant progress has been made in the development of *E*. *coli* vaccines for broilers, challenges persist. The benefits of vaccination have been demonstrated in several studies, with meta-analysis showing a positive effect of live attenuated and CpG-ODN vaccination in reducing mortality. However, further research is needed to enhance understanding of effective vaccines against colibacillosis, considering factors such as antigen selection, adjuvant choice, delivery method, and use of novel approaches.

## Supporting information

S1 Checklist PRISMA 2020 checklist. Checklist for the Preferred Reporting Items for Systematic Reviews and Meta-Analyses workflow.
(DOCX)

S1 Table Extracted metadata of studies included in the analyses of this paper. (XLSX)
